# Promotion effects of microwave heating on coalbed methane desorption compared with conductive heating

**DOI:** 10.1038/s41598-021-89193-5

**Published:** 2021-05-05

**Authors:** Zhijun Wang, Xiaojuan Wang

**Affiliations:** 1 State Key Laboratory Cultivation Base for Gas Geology and Gas Control (Henan Polytechnic University), Jiaozuo, Henan People’s Republic of China; 2 School of Safety Science and Engineering, Henan Polytechnic University, Jiaozuo, Henan People’s Republic of China; 3 Collaborative Innovation Center of Central Plains Economic Region for Coalbed /Shale Gas, Henan Province, Jiaozuo, Henan People’s Republic of China

**Keywords:** Coal, Natural gas

## Abstract

As a clean energy resource, coalbed methane (CBM) has drawn worldwide attention. However, the CBM reservoir has strong adsorption capacity and low permeability and thus requires stimulation. As a means to stimulate coalbed methane recovery, thermal injection faces geological and economic challenges because it uses conventional conductive heating (CH) to transfer heat. Realized by the conversion of the electromagnetic energy into the thermal energy, microwave heating (MH) may be a sound stimulation method. Although previous research suggested that MH had potential as a stimulation method for coalbed methane recovery, it is not clear if MH is superior to CH for enhancing coalbed methane recovery. This paper compares the effect of MH and CH on methane desorption from coal using purpose-built experimental equipment. To compare the MH and CH experimental results, the desorption temperature for each CH desorption test was set to the maximum temperature reached in the correlative MH desorption test. The results show that although the cumulative desorbed volume (CDV) of methane under MH was less than that desorbed by CH in the initial desorption stage, the final total CDV under MH for the three different power settings was ~ 12% to ~ 21% more than that desorbed by CH at the same temperatures. CH and MH both change the sample’s microstructure but MH enlarges the pores, decreases methane adsorption, promotes methane diffusion, and improves permeability more effectively than CH. Rapid temperature rise and the changes in the coal’s microstructure caused by MH were the main reasons for its superior performance. These findings may provide reference for selecting the most appropriate type of heating for thermal injection assisted coalbed methane recovery.

## Introduction

Energy resources are the backbone of countries' progress^1^. Fossil fuels, including coal, oil and natural gas, were used as a major source of energy previously. Because there is a finite amount of fossil fuels available, their depletion is a source of concern. Fossil fuels are also the main source of global warming emissions. The fact that these fuels release pollutants, such as carbon dioxide, when burned is another source of concern. The depletion of fossil fuels, global warming and greenhouse gas emissions have prompted the world to search for clean energy to replace fossil fuels, such as biomass, hydrogen, solar, nuclear, wind and hydroelectric energy^2,3^. To resolve these emissions, basically two scheme could be used, that is emission trading schemes and renewable support schemes^4,5^. Therefore, the issue to capture CO_2_ emissions emitting from the industrial processes have gained increasing concern^6–8^.

Methane is the cleanest among the conventional fossil fuels. During methane combustion, no sulfur dioxide or soot dust is emitted. The combustion products of methane are gaseous and don’t pollute the surrounding environment. Methane is nontoxic and doesn’t induce any harm to humans, animals or plants. The calorific value of methane is 1–4 times higher than that of general coal. The calorific value of 1 cubic meter of pure methane is equivalent to 1.13 kg gasoline or 1.21 kg standard coal. The advantages of using methane as a fuel where it produces a large amount of energy with lower GHG emissions compared to that of other hydrocarbons. As well as being a fuel, methane can also be converted into syngas to increase its efficiency and reduce greenhouse gas emissions. There are three main reforming routes to produce syngas from methane, which are dry reforming, steam reforming, and oxy reforming (partial oxidation)^9,10^.

Coalbed methane (CBM), a gas that coexists with the coal in most deposits, is not only a greenhouse gas and a threat to coal mine safety, it can induce gas outburst, explosions, and underground mine fires, but also a valuable nonrenewable clean energy resource^11,12^. Therefore, CBM extraction and utilization before mining can have a dual benefit by transforming a mine safety hazard into a source of clean energy. On the one hand, CBM extraction reduces the content of CBM in coal seam. The probability of gas outburst and gas explosion during the process of coal mining decreases. The safety production level of coal mine is improved. That is the first benefit. On the other hand, the extracted CBM can be used as a clean energy source and reduce the greenhouse effect, which is a second benefit. Unfortunately, the permeability of most coal seams that are likely targets for CBM exploitation is extremely low and this is especially true for coal seams in China where many of the seams are deeply buried^13–15^. This low permeability restricts commercial CBM development and production, and thus CBM reservoir stimulation is an appealing option^16,17^.

Many stimulation methods have been proposed for enhancing CBM recovery including hydro-fracturing^18^ and -slotting^19^. Hydraulic fracturing is a process which injects high-pressure liquid into an oil- or gas-bearing rock or coal formation to create fractures. It is a common technique for productivity enhancement in conventional oil and gas reservoirs. Other stimulation methods include blasting vibration^20^, CO_2_ injection enhanced coalbed methane recovery (CO_2_-ECBM)^21^, liquid nitrogen cooling^22^, and electrochemical treatments^23^. However, all these approaches face environmental and economic challenges. Hydraulics methods contaminates surface water and ground water, which has been banned in some regions^24^. Other methods have the disadvantages of special geologic conditions and high cost, and is not suitable for some reservoirs^25^. Therefore, it is necessary to discover new methods to enhance CBM recovery.

Thermal injection, which introduces heat into petroleum reservoirs to reduce the oil’s viscosity and enhance production, has been used by the petroleum industry for many years^26–29^. In recent years, thermal injection has also been applied to CBM extraction^30–32^. Studies have shown that increasing a coal seam’s temperature can decrease methane adsorption^33,34^, accelerate methane desorption and diffusion^35,36^, and increase the seam’s permeability^37,38^. Gas production can be enhanced by 58% with hot water injection^39^. According to a thermo-hydro-mechanical model, Teng et al. found that the coal’s permeability could be greatly enhanced by thermal injection^40^. Thermal injection could facilitate CBM extraction due to the enhancement of the permeability. Conventional thermal injection methods raise the temperature of the reservoir with high temperature liquid or gas using techniques like cyclic steam stimulation^41^, steam flooding^42^, or steam assisted gravity drainage^43,44^. The heat supplied by these techniques is transferred by conductive heating (CH). However, the use of these techniques in the field had revealed some of their limitations and disadvantages. These include: (1) specific geologic conditions are required for some of these techniques to be effective because it is easy for the hot liquid or steam to escape from cleats and fractures^45^. This requires that the treatment zone contains no faults, cleat apertures, fractures, caves, or large cracks penetrating the ground; (2) heat losses during transport can be significant and the temperature of the steam when it reaches the seams may be very low if the formation is very deep; (3) steam-assisted stimulation introduces a large amounts of water into the seam that can block gas seepage passages and thereby hinder CBM extraction^46^. In addition, during thermal injection, hot liquid or steam will evaporate, and vapour may spread to the surrounding non-target low-temperature areas. This can lead to rapid heat loss and significantly increase the project’s energy consumption.

To address these problems, microwave heating (MH), a non-contact physical field heat, has been proposed to replace the hot liquid or steam. Microwave heating can be a sound alternative stimulation method because it is less affected by geological condition and is capable of distributing heat through a large volume^47,48^. Compared with CH, MH has many advantages such as^49,50^: (1) it uses volumetric heating (that is it does not rely on conduction to heat the material); (2) it is noncontact, rapid, and efficient; (3) it heats selectively; (4) its operability is good and it is faster to both start and stop a heating cycle; and (5) in operation, it provides a higher level of safety than CH in part because MH can be automated. With these unique properties, MH has been applied in coal processing for operations like drying/dewatering, coking, floatation, increasing grindability, desulfurization, and enhanced coal cleaning. Up to date, some scholars have conducted the research in the field of microwave-assisted desorption and compared the desorption by conduction heating. Cherbański et al.^51^ compared the desorption kinetics and efficiencies of microwave regeneration and temperature regeneration of acetone and toluene from 13X molecular sieves. The enhancement effect of microwave on desorption is more obvious under the conditions of polar adsorbents or high resistance of heat transfer. Mao et al.^52^ compared the effect of constant power microwave heating and constant temperature microwave heating on the regeneration of spent active carbon from wheat straw and pine. The desorption rate under microwave heating with constant power was 20 times and 40 times higher than that under the microwave heating with constant temperature and the conductive heating, respectively. Fayaz et al.^53^ investigated a comparison of the desorption efficiency and the energy consumption of regeneration of microwave heating to conductive heating. They concluded that the minimum energy required under microwave heating regeneration of the adsorbent, with a 100% desorption efficiency, is 6% of the energy required under conductive heating regeneration, because the heating rate is faster and the heat loss is lower. Pi et al.^54^ compared the microwave regeneration with the conventional thermal regeneration to clarify the effects of microwave regeneration on desulfurized ACs. In comparison to the conductive heating process of 30 min, the heating speed of microwave heating is faster, and the complete regeneration can be achieved in only 4 min. But little attention has been paid to the use of microwave for CBM recovery^55–57^. Kumar et al.^58^ used X-ray computed tomography to examine a core of bituminous coal that had been irradiated with microwave energy bursts and found that new fractures were generated in the coal and the apertures of the existing fractures increased. Liu et al.^59^ investigated the pore structure of lignite after MH and discovered that the specific surface area of the irradiated coal decreased, but the coal’s average pore diameter and total pore volume increased. Wang et al.^60^ concluded that when microwave irradiation times were increased, the specific surface area of lignite samples increased, average pore diameter and total pore volume decreased, and the proportion of mesopores increased. Wang et al.^61^ examined the petrophysical response of sandstone to MH and found that after being heated, the sample’s permeability increased dramatically. Numerical simulations by Li et al. and Huang et al. indicated that high temperature difference induced by microwave irradiation is better for enhancing the permeability of coal seam^62,63^. Experimental studies conducted on coal by Hong et al., Li et al., and Hu et al. all indicated that microwave irradiation enlarged both pore size and pore throat size and also increases both then number of pores and the coal’s permeability substantially^64,65^. They concluded that MH had the potential to be applied in coalbed methane stimulation programs.

CBM recovery can be divided into two cases as on the ground and under the ground. Two microwave-assisted systems are designed for CBM recovery on the ground and CBM recovery under the ground. The two systems all consist of: (1) a CBM borehole, which is designed to host microwave apparatus and a CBM extraction tube; (2) a microwave apparatus including a microwave generator, a waveguide to coax transition, rectangular and coaxial waveguides, and an antenna; (3) a Teflon tube, which is a microwave-transparent structure interposed to safeguard the well. For CBM recovery under the ground, the microwave apparatus is placed in the roadway. The deepness of the seam has no effect on the applicability of the microwave energy. For CBM recovery on the ground, the microwave apparatus is placed on the ground. The length of coaxial waveguide depends on the depth of the seam. The depth of the coal seam has little effect on other aspects about the applicability of the microwave energy. The main possible limitation is safety hazard due to excessive temperature caused by microwave heating. The microwave heating application under the ground may be more dangerous than that on the ground. The digital temperature regulator is set as a safety interlock device; if the coal seam’s temperature becomes high enough to be dangerous, the regulator cuts off the power to the microwave generator. A methane sensor must install near the borehole for avoiding methane reaches explosion concentration^66^. In addition, the microwave source should place on the upwind.

Although a few researchers have examined the effect of microwaves on the morphology and microstructure of coal to assess the feasibility of using MH to aid CBM extraction, the actual effect of MH still needs to be verified by physical experiments before the method can be applied in the field. Coalbed methane recovery is complex and involves methane desorption, diffusion, and seepage^67,68^. Desorption is the initial stage of the whole methane migration process, which is one of the important stages to determine the efficiency of the CBM recovery. Our previous experiments have suggested that microwaves significantly promote methane desorption from coal; 1 kJ of microwave energy can cause methane desorption to increase by 0.0088 ml per gram of coal^69^. However, few reports to date have compared the effects of MH and CH on methane desorption and the coal’s microstructure. The aspects of this work that have not been already assessed in those previous reports include that comparison of desorption volume and desorption rate under MH and CH according to certain temperature standard or input energy power standard, and comparison of the comprehensive effects and their mechanisms of MH and CH on methane desorption. This is important for assessing the advantages, disadvantages, and feasibility of using MH for CBM recovery stimulation.

For this work, we developed experimental devices to investigate the effects of MH and CH on desorbing methane from coal. Changes in the coal samples’ pore structure and surface microtopography before and after MH and CH experiments were compared. The advantages and disadvantages of MH and CH on CBM recovery were documented. This work may serve as a reference for selecting the most appropriate type of heating for a thermal injection program.

## Sample preparation and experimental procedures

### Sample preparation

The coal samples used in this study were collected from the No. 21 coal seam in the Jiulishan coal mine located in Jiaozuo, Henan province, China. The No. 21 coal seam has strong adsorption capacity and low permeability, which makes it vulnerable to gas outbursts. Proximate analysis was conducted using a GF-A2000 auto proximate analyzer and according to the Chinese National Standard GB/T 212-2008 with the following results: moisture (2.1%), ash content (12.40%), and volatiles (8.62%). This standard is different from that used in the literatures^70,71^. The coal samples used for this MH study were collected from the same location and were then ground to powders and sieved with metal sieves in the laboratory to produce specimens of 0.5–1 mm grain size. The specimens were then placed in a drying oven at 378.15 K to dry. After drying, the prepared specimens were stored in a dry environment. So that the results of the different experiments would be comparable, fresh splits of these dried coal specimens were used in each experiment.

### Experimental procedures

**Figure 1:**
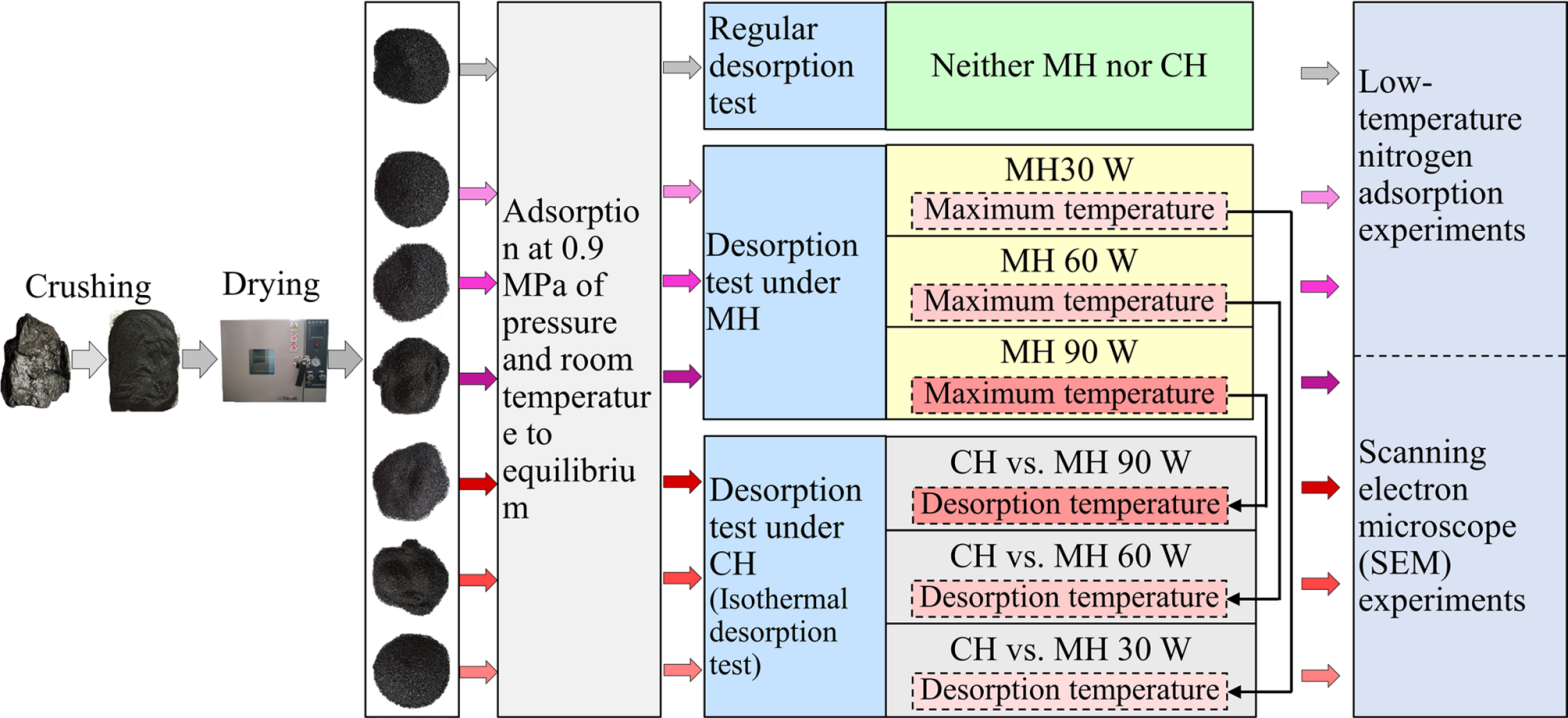
Flow chart showing desorption test experimental procedures.

For this work, three different types of desorption tests were carried out, a room temperature desorption test (neither MH nor CH were applied), desorption tests under MH, and desorption tests under CH (Fig. 1). To make the tests as comparable as possible, the coal samples used for each test had the same mass, and the adsorption stage of each experiment were all done under the same pressure (~ 100 kPa = 1 atm) and at room temperature (29 °C = 302.15 K). For the room temperature desorption test, desorption took place naturally without external interference; neither MH nor CH were applied. For the MH desorption tests, microwaves with a specific power were loaded on the coal samples during desorption. According to the work of Cai and his coworkers, coal samples will not pyrolize at temperatures below 573 K^37^. To avoid pyrolysis, microwave power for the experiments was set at 30, 60, or 90 W. The corresponding MH tests were called the MH 30 W, MH 60 W, and MH 90 W tests. For the MH coal desorption tests, the temperature of the coal sample gradually increased during the course of the experiment. Because heat transfer by conduction takes time, it is very difficult to replicate the temperature changes that occur under the MH experimental conditions when heating by conduction. Therefore, the CH desorption tests were run as isothermal experiments. To compare the MH and CH experimental results, the desorption temperature for each CH desorption test was set to the maximum temperature reached in the correlative MH desorption test. The CH testing oven was preheated to that temperature before the sample was introduced. The CH desorption tests corresponding to the MH 30 W, MH 60 W, and MH 90 W tests were called CH vs. MH 30 W, CH vs. MH 60 W, and CH vs. MH 90 W. According to work by Tang and his coworkers, methane desorption increases with increasing temperature^35^. Obviously the desorption effects of the temperatures induced by MH will be weaker than desorption caused by the constant heat transfer during the CH tests. This difference is unavoidable. The experimental conditions are listed in Table 1.

**Table 1:** Microwave and conductive heating desorption test experimental conditions.

Test mode	Experimental condition	Adsorption equilibrium pressure (MPa)	Room temperature (K)	Microwave power(W)	Test duration (min)	Desorption temperature
Regular desorption test	Neither MH nor CH	0.9	302.15	–	180	Room temperature
Desorption test under MH	MH 30 W	0.9	302.15	30	180	Real temperature induced by MH 30 W
	MH 60 W	0.9	302.15	60	180	Real temperature induced by MH 60 W
	MH 90 W	0.9	302.15	90	180	Real temperature induced by MH 90 W
Desorption test under CH	CH vs. MH 30 W	0.9	302.15	–	180	Maximum temperature caused by MH 30 W
	CH vs. MH 60 W	0.9	302.15	–	180	Maximum temperature caused by MH 60 W
	CH vs. MH 90 W	0.9	302.15	–	180	Maximum temperature caused by MH 90 W

### Experimental apparatus

**Figure 2:**
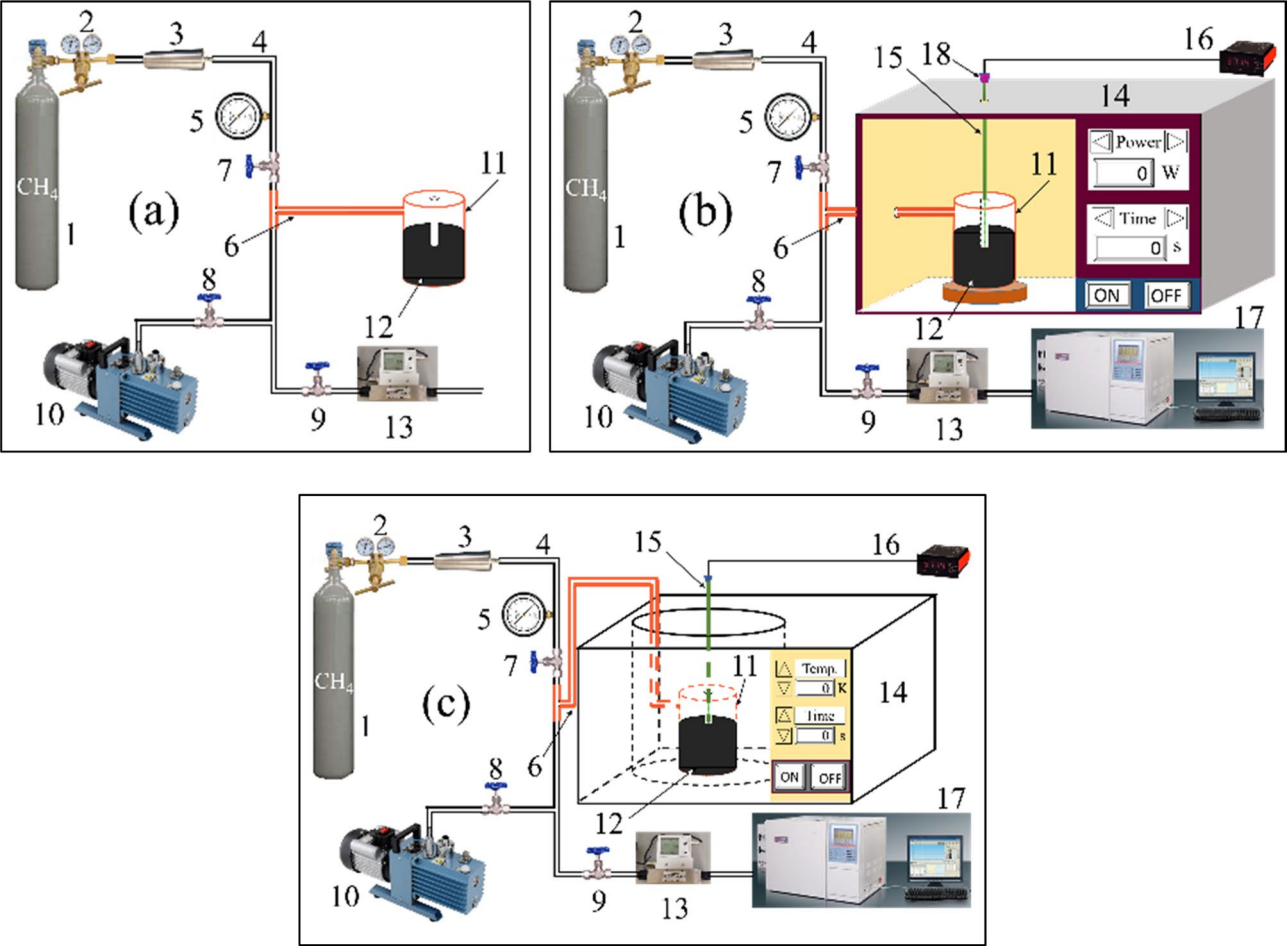
Experimental apparatus used for measuring methane desorption: (**a**) at room temperature desorption; (**b**) under MH; (**c**) under CH. 1—Gas container (99.99% CH_4_), 2—Reducing valve, 3—Buffer tank, 4—Steel pipe, 5—Precision pressure gauge, 6—Teflon pipe, 7–9—Valves, 10—Vacuum pump, 11—Teflon canister, 12—Coal sample, 13—Flow meter, 14 on (**b**)—Microwave generator, 14 on (**c**)—Ultra-thermostat, 15—Thermocouple, 16—Temperature indicator, 17—Gas chromatograph, 18—Filter capacitor.

The three different types of desorption experiments were carried out on three types of experimental systems. The room temperature desorption, MH desorption, and CH desorption experiments were conducted on the equipment shown in Fig. 2a–c, respectively. The MH methane desorption testing system was assembled in-house. It consists of a microwave generator with a frequency of 2450 ± 25 MHz, a teflon canister, a gas container, a buffer tank, a pressure gauge, a temperature measuring unit, a vacuum pump, a flow meter, a gas chromatograph, three valves, a number of teflon pipes, and some steel pipes. The microwave generator’s power output is adjustable from 0 to 900 W and irradiation time is controlled by a timer. A microwave agitator is attached in the microwave generator. The microwave agitator can excite more electromagnetic field modes in the cavity. The positions of the wave peak and nodes of the superposed standing wave field are constantly shifted. It can improve the uniformity of the microwave field distribution in the cavity and make the microwave heating more uniform. The adsorption gas used in the MH experiments was high purity 99.99% methane. A drivepipe is fixed in the mouth of the teflon canister to hold a thermocouple that measures the temperature in the center of coal the sample during an experiment in real time. The temperature-measuring unit consists of a K-type thermocouple, a digital temperature regulator/display and a filter capacitor. The filter capacitor was added to the end of the thermocouple to eliminate high frequency interference and “sparking” in the microwave generator that would otherwise result from the device. A gas chromatograph was used to measure the composition of the desorbed gas during the experiments. The CH desorption experimental system was the same as the system used for the MH experiments except that an oven controlled by a thermostat is substituted for the microwave generator.

### Dielectric measurements

In general, the ability of a material to absorb microwave energy is directly related to its dielectric properties. The dielectric properties of a material are normally represented by complex relative permittivity:

1 $$\varepsilon_{r} = \varepsilon^{\prime} - j\varepsilon^{\prime\prime}$$

where *ε*_*r*_ is complex relative permittivity, *ε*′ is the relative dielectric constant that examines the capacity of a material to store electromagnetic energy, *ε*′′ is the relative dielectric loss factor that characterizes the conversion of electromagnetic energy into thermal energy in the material.

For measurements of dielectric properties, the cavity perturbation method was used in the study. The coal sample was first ground to powders having size less than 175 μm and then dried for 24 h. The dielectric constant *ε*′ and loss factor *ε*′′ of the coal powders were measured by the measurement system, which was mainly constituted by a resistance furnace, an Agilent N5230A vector network analyzer and a cylindrical TM_0n_0 resonant mode cavity with a diameter of 580 mm and a height of 50 mm. A thermostatic water bath was used to heat the sample to the preset temperature and the cavity was employed to detect the above mentioned cavity response differences caused by sample at frequency of 2.45 GHz, which were subsequently recorded in the Agilent N5230A vector network analyzer (dynamic range: 108 dB, trajectory noise: < 0.004 dB and measurement speed: < 4.5 μs/point). By obtaining the differences in the microwave cavity response, the complex electric susceptibility of the sample was computed and used for calculation of corresponding dielectric parameters.

### Low-temperature nitrogen adsorption and SEM experiments

Low-temperature nitrogen adsorption is a common way to determine a coal sample’s porosity. In this study, an automatic surface area and pore analyzer (an ASAP 2020, Micrometrics, Norcross, GA, USA) was used to determine sample porosities before and after the MH and CH experiments. Nitrogen adsorption/desorption isotherms were measured at 77 K for the relative pressure (P/P_0_) range 0.01–0.99. The surface microtopographies of the coal samples before and after the MH and CH were also examined with a scanning electron microscope (SEM) (a Quanta 250 SEM, FEI Company, Hillsboro, OR, USA).

## Results

### Dielectric characterization and penetration depth

**Figure 3:**
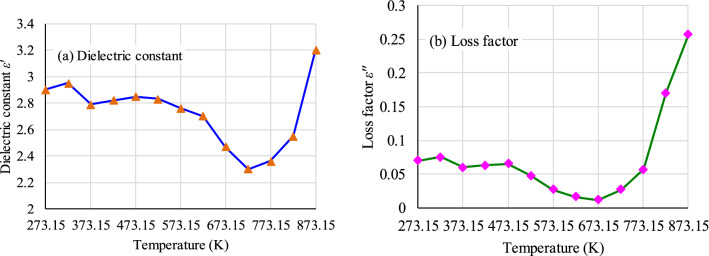
Variations of dielectric constant and loss factor of coal sample versus temperature at 2.45 GHz.

The dielectric constant and loss factor are the most important parameters that control the microwave power absorption of a material. Figure 3 presents the temperature dependences of these parameters of the coal sample. Their results showed that these dielectric parameters present a “U” trend with temperature increasing. Their minimum values appear between 673.15 K and 723.15 K. These dielectric parameters gradually decrease with increasing temperature below 473.15 K due to dewatering. From 523.15 to 723.15 K, these parameters decrease slightly due to the loss of volatiles in the coal. When the temperature exceeds about 723.15 K, obvious chemical changes will occur in the coal body, mainly including the beginning of chemical bond breakage and the macromolecular structure change. Because of devolatilization, decomposition and carbonization, a few of oxygen functional groups are removed, such as aliphatic, phenolic and carboxylic groups. These dielectric parameters rapidly increase.

As microwave penetrates into a material, its amplitude diminishes. This attenuation can be expressed by the penetration depth, *D*_*p*_ (the depth at which the power flux falls to 1/e of its surface value)^72^. *D*_*p*_ is given by

2 $$D_{p} = \frac{C}{{2{\uppi }f\sqrt {2\varepsilon^{\prime}} \left[ {\sqrt {1 + \tan^{2} \left( {\frac{{\varepsilon^{\prime\prime}}}{{\varepsilon^{\prime}}}} \right)} - 1} \right]^{1/2} }}$$

where *C* is the speed of light, m/s; *f* is the frequency, Hz. *D*_*p*_ varies from metres to millimeters depending on the frequency, temperature, chemical composition and microstructure^73^. *D*_*p*_ in coal is on a scale of meters because of the low *ε*′^51^.

**Figure 4:**
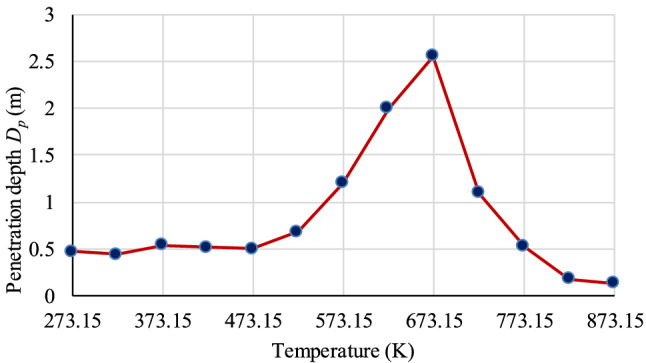
Microwave penetration depth of coal sample versus temperature at 2.45 GHz.

Based on the test results of the dielectric parameters, the penetration depth can be calculated by Eq. (2), shown in Fig. 4. It is noticed that the depth initially increases and then declines with increasing temperature. The microwave penetration depth of the coal presents a depth peak at approximately 673.15 K. At this temperature point, *ε*ʹ = 2.47, *ε*″ = 0.012, *f* = 2.45 GHz, and *D*_*p*_ = 2.55 m. The volume of the treatment is approximately a cylinder. If the thickness of the coal seam is 3 m (the thickness of the common coal seam), the estimated volume that can be treated is 61.3 m^3^. The estimated power consumption to treat certain volume is 1.34 × 10^8^ kJ (heat capacity of coal 4.4 kJ/(kg K), coal density 1.38 g/cm^3^, initial temperature 313.15 K). The influence range of microwave will be even larger because of heat transfer.

### Microwave heating and methane desorption

#### Desorption volume

**Figure 5:**
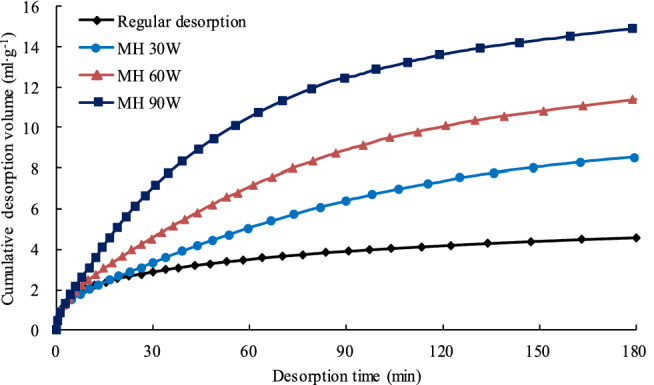
Cumulative desorbed volume of methane vs. time curves for MH and room temperature coal desorption tests.

The cumulative desorbed volume (CDV)–time curves for gas desorbed during the room temperature desorption test and the MH desorption tests are shown in Fig. 5. From Fig. 5, it is clear that more methane desorbs from the coal during MH than desorbs at room temperature. The total cumulative desorbed volumes (TCDV) for the MH 30 W, MH 60 W, and MH 90 W tests were 1.87, 2.49 and 3.26 times larger than TCDV during the room temperature desorption test. It is clear that MH can greatly increases the total amount of methane desorbed from the coal. Figure 5 also shows that the higher the microwave power is, the larger the TCDV of methane.

#### Desorption rate

**Figure 6:**
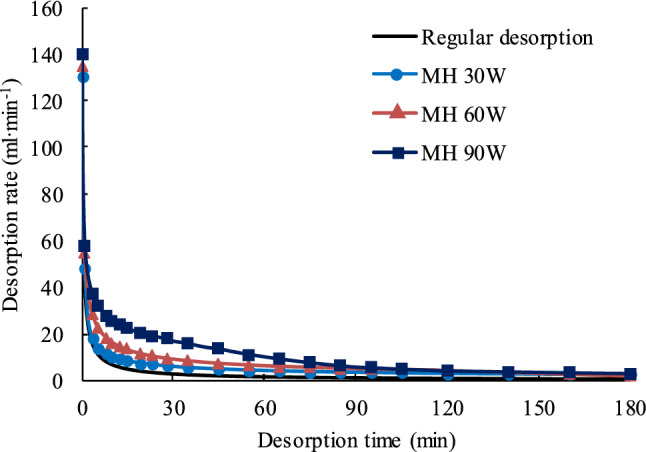
Graph showing desorption rates for MH and room temperature desorption tests.

Figure 6 compares methane desorption rates for the MH and room temperature desorption tests during the 180 min test duration. From Fig. 6 it can be seen that desorption rates are high for both room temperature and MH desorption tests at the initial test stage, and then the rate decreases until it eventually stabilizes. Even though the rate decreases, the MH desorption rate is higher than the room temperature rate for the entire 180 min test period. In addition, the higher the microwave power is, the greater the desorption rate. These test results show that MH with low power can improve the methane desorption rate and even mitigate desorption rate attenuation to some extent, however, MH does not change the tendency of the methane desorption rate to decline.

#### Temperature changes

**Figure 7:**
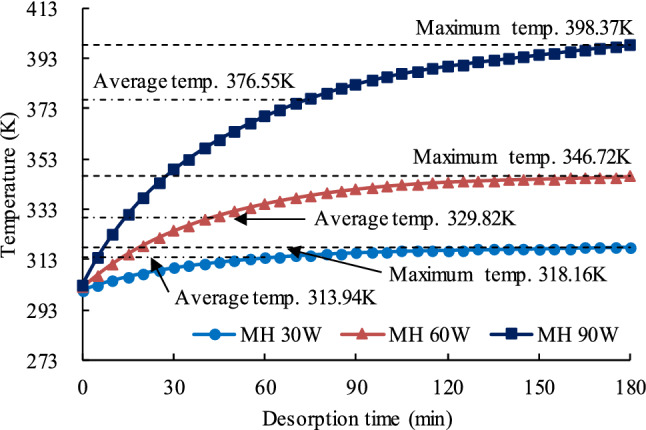
Graph showing temperature vs. time for the coal samples during the three MH desorption tests.

Coal is a typical dielectric material. When coal is placed in a microwave field, the microwaves pass through the coal and a portion of the microwave energy is absorbed and converted into thermal energy owing to the inner friction of particles in the coal. As a result, the coal is heated and its temperature increases. In our experiments, the temperature in the center of the coal samples subjected to microwaves was measured with a thermocouple (Fig. 2b, item 15). The temperatures in the samples show the same general trend for all three microwave power settings. The temperature first increases quickly but then the rate of increase slows. This trend can be divided into three stages. In the first stage, the MH causes the temperature to rise quickly and the rate of temperature increase is as much as 1.735 K/min. In the second stage the temperature increases slower because the heat extracted by desorption becomes more significant. The rate at which the temperature rises decreases the dielectric loss from the coal, the rate of temperature increase declines with the decreased dielectric loss. In stage three, the coal’s temperature approaches a constant value. As the coal sample’s temperature increases, both its capacity to absorb heat and the amount of heat it dissipates stabilize and the heat exchange reaches equilibrium. The final coal temperatures after 180 min of MH at 30, 60, and 90 W were 318.16 K, 346.72 K, and 398.37 K, respectively. Integrating the time–temperature curves plotted on Fig. 7 shows that the average temperatures of the coal samples during desorption for the MH 30 W, MH 60 W, and MH 90 W tests were 313.94 K, 329.82 K, and 376.55 K, respectively.

### Microwave heating–conductive heating methane desorption comparison

**Figure 8:**
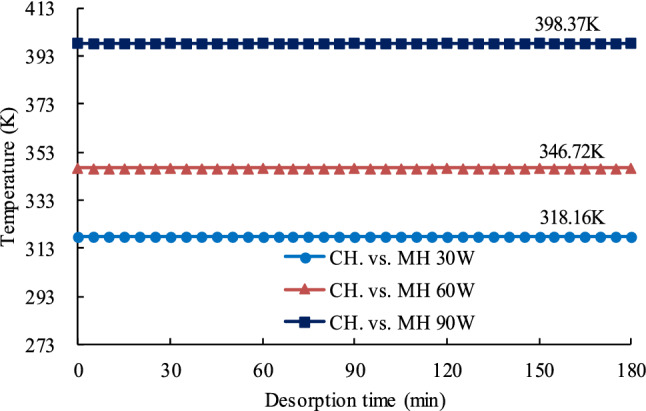
Coal sample temperatures during CH desorption tests.

As shown on Fig. 7, the maximum temperatures reached during the MH 30 W, MH 60 W, and MH 90 W tests were 318.16 K, 346.72 K, and 398.37 K, respectively. Therefore, to make the test conditions as similar as possible, the three CH isothermal tests, CH vs. MH 30 W, CH vs. MH 60 W, and CH vs. MH 90 W, were conducted at those same three temperatures. Coal sample temperatures during the CH desorption tests are shown in Fig. 8.

**Figure 9:**
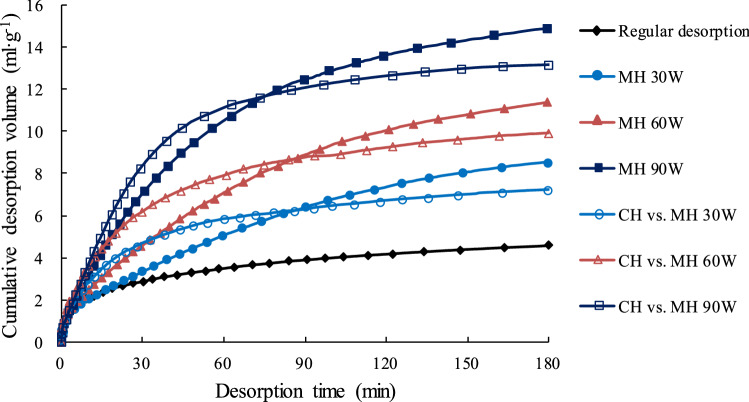
Comparisons of CDV for the MH and CH experiments.

Curves comparing the CDVs for the MH and CH tests are shown in Fig. 9. From Fig. 9, it can be seen that all MH and CH CDVs greatly exceed the room temperature CDV. The higher the final desorption temperature, the greater the amount of gas desorbed. For the same maximum temperature, the total cumulative desorbed volume (TCDV) from the MH tests was higher than that from the CH tests even though the MH CDVs were lower that the MH CDVs in the initial desorption stage. After the tests were completed, the MH TCDVs with microwave power levels of 30, 60, and 90 W were 20.8%, 18.4%, and 11.7% higher than those under the corresponding CH TCDVs.

**Figure 10:**
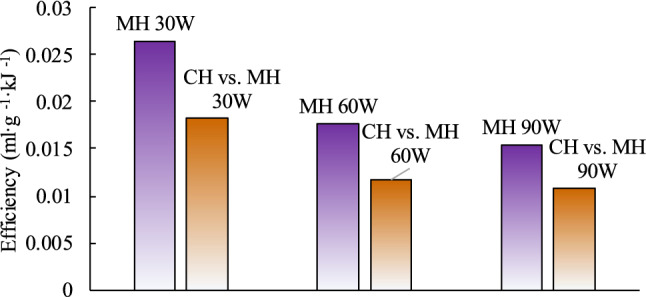
Comparison of the energy utilization efficiency under MH and CH.

Energy input is the main factor that causes more methane to be desorbed during the MH and CH tests than is desorbed at room temperature. The amount of total energy input used during the MH and CH tests is explored. The total energy input values are 324 kJ, 648 kJ, 972 kJ, 394 kJ, 812 kJ and 1236 kJ for MH 30 W, MH 60 W, MH 90 W, CH vs. MH 30 W, CH vs. MH 60 W, and CH vs. MH 90 W respectively. The energy utilization efficiency in the two heating modes can be calculated by:

3 $${\text{Efficiency}} = \frac{{{\text{Total }}\;{\text{cumulative}}\;{\text{ desorbed }}\;{\text{volume}}}}{{{\text{Total }}\;{\text{energy}}\;{\text{ input}}}}$$

where efficiency denotes the total cumulative desorbed volume caused by 1 kJ of the total energy input, ml g^−1^ kJ^−1^. Figure 10 shows the comparison of the energy utilization efficiency in the two heating modes. It demonstrate that the energy utilization efficiency under MH is larger than that under CH.

**Figure 11:**
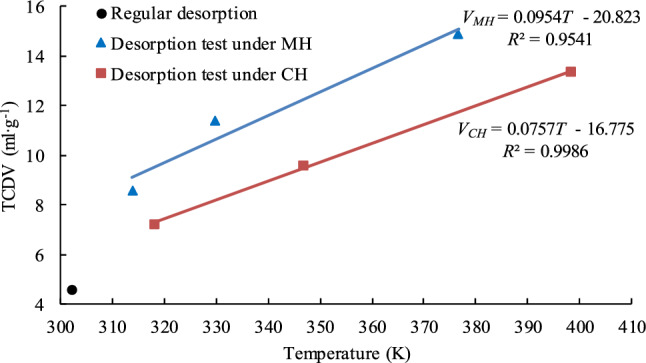
Linear relationship between TCDV and temperature. The lines and equations in the graph are the linear regressions fit to the TCDV values and the final temperatures shown in Fig. 9.

Furthermore, the relation between TCDV and the desorption temperature was obtained by fitting a linear regression to the TCDV values and the final temperatures shown in Fig. 9. The linear regression equations for the MH and CH test data, Eqs. (4) and (5), are below and the data fit by the regressions are shown in Fig. 11.

4 $$V_{CH} = \, 0.0{757}T - { 16}.{775} \;\;\;\;\;\;\;\;\; R^{{2}} = \, 0.{9986}$$

where *V*_*CH*_ is the TCDV for coal samples heated by CH, ml/g; *T* is the CH desorption temperature, and K.

5 $$V_{MH} = \, 0.0{954}T - { 2}0.{823} \;\;\;\;\;\;\;\;\;\; R^{{2}} = \, 0.{9541}$$

where *V*_*MH*_ is TCDV for coal samples heated by MH, ml/g; *T* is the average MH desorption temperature, K.

From the CH equation, Eq. (4), if the desorption temperature is increased by one degree, the TCDV will increase 0.0757 ml/g. From the MH equation, Eq. (5), if the average desorption temperature is increased by one degree, the TCDV will increase by 0.0954 ml/g, 26.0% more than the increase from an equivalent CH temperature increase. Solving Eqs. (4) and (5) for temperatures between 310 and 410 K shows that the MH TCDV will be between 26.9 and 30.8% larger than the CH TCDV. These percentages are for the CH desorption temperature being equal to the maximum MH desorption temperature. If the CH desorption temperature is assumed to be the average MH desorption temperature, the percentage differences in the TCDVs would be even larger.

The above calculations suggest that MH can cause methane to be desorbed from coal better than CH and MH has better energy utilization efficiency. It has also been proposed that there are other, non-thermal effects caused by MH that may promote methane desorption. Some of these effects are discussed below.

### Coal porosity and methane adsorption/desorption

If the relative condensation and evaporation pressures are different, the liquid nitrogen adsorption and desorption curves will form a desorption loop due to capillary condensation. According to their shape, the pores in coal can be divided into three types^74^. These type differ in that some type can produce desorption loops, some cannot. Type I pores are open pore and provide good permeability. They include tubular pore, open at both ends, and parallel plate pore with four open sides. This type of pore can produce adsorption loops. Type II pores are airtight pore, closed at one end, and include closed tubular pores, parallel plate pores, wedge-shape pores, and tapered pores. All the Type II pores are closed at one end and this type of pore will not produce an adsorption loop. Type III pores are a special form of pore called an ink-bottle-shaped pore because their form is a relatively large pore with only a narrow neck for an opening. Although this type of pore is closed at one end, it can generate an adsorption loop and these loops have an inflection point with a sharp decline in the desorption branch of the loop.

Research has shown that coals with poor gas permeability cannot produce adsorption loops or can only produce adsorption loops with small areas^75,76^. Coals with good gas permeability can produce adsorption loops that surround large areas. Apparently, the shape of the adsorption loop can reflect both the coal’s pore structure and the shapes of some pores; both these properties play a major role in methane adsorption. Pore structure and pore shape may also be useful for evaluating the coal’s permeability.

**Figure 12:**
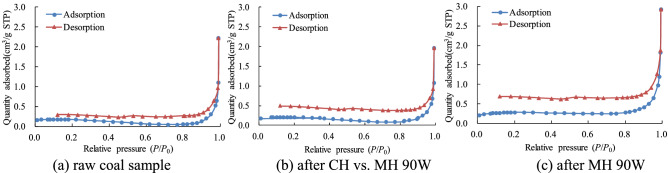
Adsorption/desorption isotherms for a sample of raw coal, a coal sample after CH vs. MH 90 W, and a coal sample after MH 90 W.

Figure 12 shows the results of the low-temperature nitrogen adsorption experiments. The figure shows adsorption and desorption isotherms for a sample of raw coal and for two samples after MH 90 W and CH vs. MH 90 W heating. As can be seen on Fig. 12a, the area inside the raw coal adsorption loop is very small probably because the pores were mainly closed at one end and impermeable. Gas flow resistance in the coal seam from which the sample was taken was high and the permeability was low. The adsorption loop area for the coal sample after CH vs. MH 90 W heating, Fig. 12b, is larger probably because the diameters of the small and medium-sized pores were increased. The number of open pores in sample CH vs. MH 90 W probably increased and the number of closed pores decreased so permeability was improved. After the MH 90 W heating of the MH sample, the sample’s adsorption loop area is even larger (Fig. 12c). This indicates that MH can further increase the proportion of open pores, reduce gas flow resistance in the sample even more, and improve the sample’s permeability to a greater extent than these properties can be augmented by CH.

The specific surface areas and average pore sizes for the coal samples used for this study are listed in Table 2. The data in Table 2 show that relative to the raw coal sample, the specific surface area decreased by 4.81% after CH vs. MH 90 W heating and by 41.1% after MH 90 W heating. The average pore size after those two tests were conducted increased by 13.0% and 36.9%. Generally speaking, the specific surface area decreases, the average pore size increases, the percentage of open pores increases, and the adsorption capacity decreases^77,78^. The data show that MH increased the percentage of open pores and decreased the adsorption capacity more than CH did. In short, CH and MH both change the pore structure to some extent and MH increases average pore size, decreases the methane adsorption capacity, enhances methane desorption, promotes diffusion, and improves permeability more effectively than CH.

**Table 2:** Specific surface areas and average pore sizes for raw and heated coal samples used in this study.

Experimental condition	Raw coal sample	After CH vs. MH 90 W	After CH vs. MH 90 W
Specific surface area (m^2^/g)	0.48705	0.46362	0.28673
Average pore size (nm)	17.153	19.377	23.480

### Coal sample surface microtopographic changes

**Figure 13:**
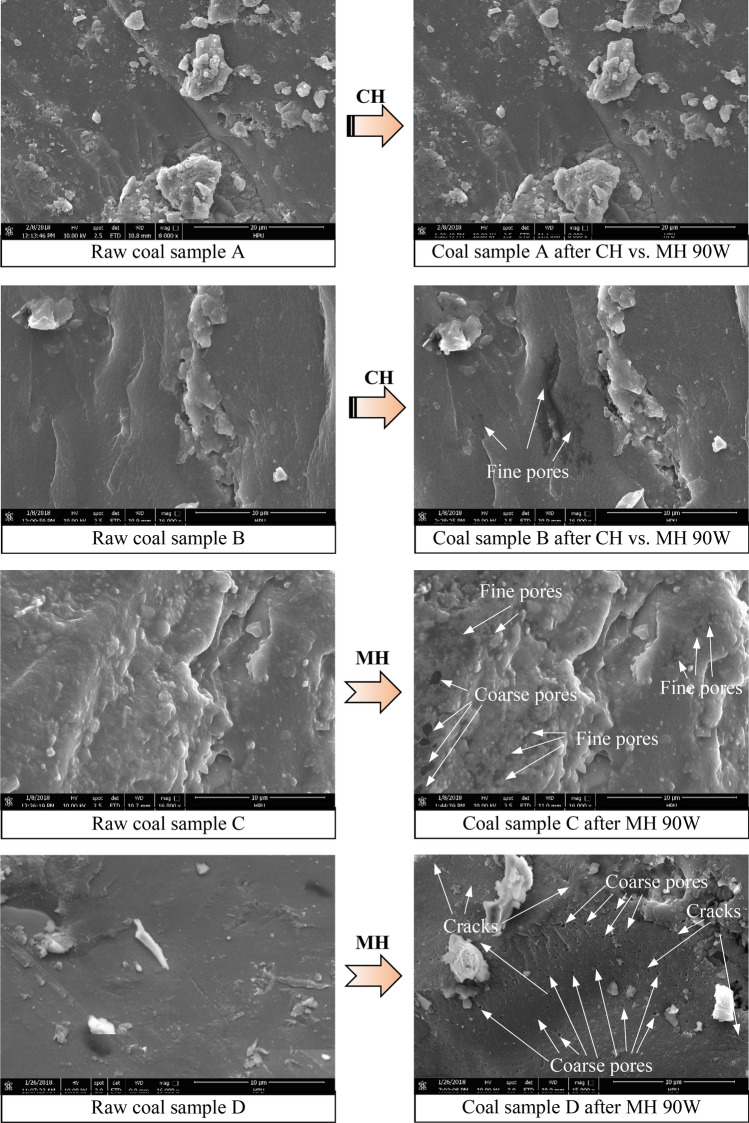
Representative SEM images showing changes on the surfaces of coal samples before and after MH and CH.

To compare the effect of MH and CH on the surface microtopographies of the coal samples, a number of SEM images showing small areas of samples when they were raw coal samples and after the sample had been heated by CH vs. MH 90 W or MH 90 W were generated. Representative examples of those SEM images are shown in Fig. 13. When inspecting the mages in Fig. 13, it can be seen that the surfaces of the raw coal samples are relatively smooth and few pores or cracks are visible. After coal sample A was heated by CH vs. MH 90 W, the sample’s surface did not change appreciable but the surface of coal sample B after CH vs. MH 90 W was not the same. On sample B some isolated fine pores are visible in the center of the image. After MH 90 W heating, it is apparent that the micro-topographies of coal samples C and D are quite different. On the coal sample C image, both fine and coarse pores are visible across most of the sample’s surface and on the image of coal sample D, there are numerous coarse pores and a few cracks clearly visible. Different coal samples have different responses to MH and CH, but in general, it can be seen that for the same temperature, MH has a more pronounced effect on the coal’s microstructure than CH does. This may be because MH causes the temperature in the coal to rise much more rapidly than when the coal is heated by CH. High temperatures cause the moisture, volatiles, and micromolecular organic matter in the pores in the coal to melt or vaporize and this raises the pressure in the pores. The increased pressure enlarges pre-existing pores and can also cause new pores or holes to form. The higher the rate of temperature increase, the faster the gas pressure rises and the more significant the pore enlarging effect. Microwave heating gives rise to volumetric heating with faster heating rates than CH so MH enlarges the pores more significantly than CH.

## Discussion

To further compare and analyze the differences between MH and CH desorption, the differences between the MH CDVs and the corresponding CH CDVs over the course of the experiment were calculated by subtracting one CDV from the other. The equation for this calculation is:

6 $$Q\left( t \right)_{{{\text{MH}}}} - Q\left( t \right)_{{{\text{CH}}}} = Q_{diff} \left( t \right)$$

where *Q*(*t*)_MH_ is CDV at time *t* under MH, ml/g; *Q*(*t*)_CH_ is CDV at time *t* under CH, ml/g; *Q*_*diff*_(*t*) is the difference between *Q*(*t*)_CH_ and *Q*(*t*)_MH_ at time *t*, ml/g; *t* is the desorption time, min.

**Figure 14:**
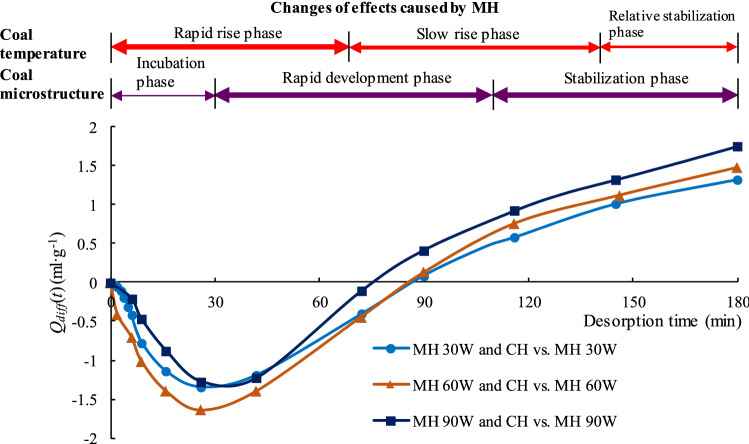
Graph showing the difference between MH CDV and CH CDV vs. desorption time. The widths of the horizontal lines above the graph are proportional to the intensity of the effect.

The changes in *Q*_*diff*_ over the course of the 180 min desorption experiments are shown in Fig. 14. The figure shows that how MH affects the methane desorption in coal is different in different stages. When *t* = 0, the temperature of the coal samples in the CH experiments was essentially the same as the maximum temperature for that experiment but the temperature of the coal samples in the MH experiments was room temperature (302.15 K). At *t* = 0, the difference in temperature between the coal samples in the CH experiments and those in the MH experiments was the largest. In the MH experiments, the temperature of the coal samples increased rapidly from room temperature. At *t* = 35 min, the temperature had risen to approximately half of the maximum temperature for that experiment (Fig. 7). However, the average temperature of the coal sample was relatively low at this stage and the changes in the coal’s microstructure were still developing under the effects of the rapidly rising temperature. As is well known, methane desorption from coal is endothermic and a higher temperature can provide energy for this reaction and promote methane desorption and release. According to research results by He et al.^79^, the rate at which the temperature increases has an important effect on desorption. The higher the rate, the faster the desorption and the greater the amount of gas desorbed. In the initial stage (0–35 min), the average temperature of the coal sample is still relatively low. The rapid volumetric heating and selective heating caused by MH began to change the coal’s microstructure but these changes are still in a preliminary development stage. Therefore, the rate of the temperature rise in this stage still plays a major role in promoting methane desorption.

After 35 min, the rate of temperature rise began to fall but the coal’s temperatures continued to rise, the pore structure began to change, and microfissures began to develop rapidly. At this stage, the channels for methane desorption and diffusion began to become unobstructed. At around 85 min, *Q*_*diff*_ (*t*) was close to zero, MH CDV had overtaken MH CDV, and the rate of temperature rise had fallen further. The faster desorption rate under CH in the early stage of the process, say from time 0 min to a time between 60 and 90 min (depending on the power level) in Fig. 9, was due to the higher temperature under CH over this time interval, as clearly shown in Figs. 7 and 8. This is why *Q*_*diff*_ values are negative between 0 and 90 min in Fig. 14. At 110 min, further alterations to the coal’s microstructure had essentially ceased. From 110 min to the end of the experiment, both the temperature and the coal’s microstructure remained relatively stable and the amount of gas being desorbed was relatively constant. At the end of the process, when the measured temperatures under the two heating modes is approximately the same, the effect of MH on the pores structure prevails, desorption becomes faster under MH and the TCDV is higher. Eventually, the MH TCDV was 11.7%–20.8% more than the CH TCDV.

The changes in temperature and of the coal’s microstructure during desorption under MH can be divided into several separate phases (Fig. 14). The temperature changes can be divided into three phases, a rapid temperature rise (0–70 min), a slow temperature rise (71–140 min), and relatively stable phase (141–180 min). The microstructural changes can also be divided into three phases, a slow development phase (0–35 min), a rapid development phase (35–110 min), and a stabile phase (110–180 min). The comprehensive effect of MH on methane desorption is the combined effects of these two chains of events in time and space.

## Conclusions

At the frequency of 2.45 GHz, the dielectric parameters present a ‘‘U’’ trend with temperature increasing. Microwave penetration depth initially increases and then declines with increasing temperature. The peak of the penetration depth is 2.55 m at approximately 673.15 K. The estimated volume that can be treated is 61.3 m^3^.

It is clear that microwave heating (MH) promotes methane desorption from coal. The higher the MH power, the more microwave energy is transferred, the higher the methane desorption rate and the larger total volume of methane desorbed. The total cumulative desorbed volumes for the MH 30 W, MH 60 W, and MH 90 W tests were 1.87, 2.49 and 3.26 times larger than those during the room temperature desorption test.

Conductive heating (CH) also promotes methane desorption from coal and the higher the CH temperature, the higher the methane desorption rate and the larger total volume of methane desorbed.

At the same maximum temperature, the total volume of methane desorbed by MH is larger than the volume desorbed by CH, although the volume of gas desorbed by MH is less than that desorbed by CH in the initial desorption stage. The final total cumulative desorbed volumes under MH for the three different power settings were ~ 12% to ~ 21% more than those desorbed by CH at the same temperatures. This implies that MH can promote methane desorption more effectively than CH can.

CH and MH both change the microstructure of the coal to some extent but MH enlarges the pores, promotes methane diffusion, and improves permeability more effectively than CH.

MH primarily promotes methane desorption by raising the coal’s temperature rapidly and changing the coal’s microstructure. In the early stage, methane desorption is mainly enhanced by the rapid increase in temperature but in the middle and later stages, the enhanced desorption is largely due to changes in the coal’s microstructure.

According to the comparison of the total cumulative desorbed volume, energy efficiency and comprehensive performance, MH has better performance in promoting methane desorption than CH. These findings can provide important reference for selecting the most appropriate type of heating for a thermal injection program.

The research on the performance of MH on treating large bulk coal samples will be carried out in the future, so as to provide the reference for the field application of MH.
